# Ultrastructure of the gill ciliary epithelium of *Limnoperna fortunei* (Dunker 1857), the invasive golden mussel

**DOI:** 10.1186/s40850-022-00107-y

**Published:** 2022-01-17

**Authors:** Erico Tadeu Fraga Freitas, Amanda Maria Siqueira Moreira, Rayan Silva de Paula, Gabriela Rabelo Andrade, Marcela David de Carvalho, Paulo Santos Assis, Erika Cristina Jorge, Antônio Valadão Cardoso

**Affiliations:** 1 Centro de Bioengenharia de Espécies Invasoras de Hidrelétricas (CBEIH), 31035-536 Belo Horizonte, MG Brazil; 2 Centro de Microscopia, Universidade Federal de Minas Gerais (UFMG), 31270-901 Belo Horizonte, MG Brazil; 3 Universidade Federal de Ouro Preto (UFOP), FIMAT, 35400-000 Ouro Preto, MG Brazil; 4 Instituto de Ciências Biológicas, Universidade Federal de Minas Gerais (UFMG), 31270-901 Belo Horizonte, MG Brazil; 5 Companhia Energética de Minas Gerais SA (CEMIG), 30190-131 Belo Horizonte, MG Brazil; 6 Escola de Design, Universidade do Estado de Minas Gerais (UEMG), 30140-091 Belo Horizonte, MG Brazil

**Keywords:** Golden mussel, Invasive species, Mussel gill, Suspension-feeding, Branchial epithelium, Electron microscopy

## Abstract

**Background:**

*Limnoperna fortunei* is a freshwater bivalve mollusc originally from southern Asia that invaded South America in the 1990’s. Due to its highly efficient water pumping and filtering, and its capacity to form strong adhesions to a variety of substrates by byssus thread, this invasive species has been able to adapt to several environments across South America, causing significant ecological and economic damages. By gaining a deeper understanding of the biological and ecological aspects of *L. fortunei* we will be able to establish more effective strategies to manage its invasion. The gills of the mollusc are key structures responsible for several biological functions, including respiration and feeding. In this work, we characterized the ultrastructure of *L. fortunei* gills and its ciliary epithelium using light microscopy, transmission and scanning electron microscopies. This is the first report of the morphology of the epithelial cells and cilia of the gill of *L. fortunei* visualized in high resolution.

**Results:**

The analysis showed highly organized and abundant ciliary structures (lateral cilia, laterofrontal cirri and frontal cilia) on the entire length of the branchial epithelium. Mitochondria, smooth endoplasmic reticulum and glycogen granules were abundantly found in the epithelial cells of the gills, demonstrating the energy-demanding function of these structures. Neutral mucopolysaccharides (low viscosity mucus) were observed on the frontal surface of the gill filaments and acid mucopolysaccharides (high viscosity mucus) were observed to be spread out, mainly on the lateral tract. Spherical vesicles, possibly containing mucus, could also be observed in these cells. These findings demonstrate the importance of the mucociliary processes in particle capture and selection.

**Conclusions:**

Our data suggest that the mechanism used by this mollusc for particle capture and selection could contribute to a better understanding of key aspects of invasion and also in the establishment of more efficient and economically viable strategies of population control.

**Supplementary Information:**

The online version contains supplementary material available at 10.1186/s40850-022-00107-y.

## Background

Biological invasions of alien animals and plants are one of the most critical threats to biodiversity in aquatic ecosystems. Among the known invasive species, bivalve molluscs are responsible for causing both significant environmental and economic damages [1]. *Limnoperna fortunei* (Dunker 1857) and *Corbicula fluminea* (Müller 1774) are among those bivalves known to have become established invaders in South America [2, 3]. *Limnoperna fortunei* is a bivalve belonging to the family Mytilidae (subclass Pteriomorphia and order Mytiloida) and is originally native to Southeast Asia (including China and South Korea) [4]. The arrival of this invasive mollusc in South America occurred in the early 1990’s, possibly transported by ballast waters from cargo ships originating in Asia due to the increase in trade routes between the two continents [5].

*Limnoperna fortunei* can inhabit waters with a wide range of temperatures and salinity and cope with long periods of air exposure [6, 7]. Understanding the morphological aspects of *L. fortunei* structures is key to further understanding these biological invasions. Some recent studies focused on the morphology and function of the cilia on the *L. fortunei* foot, used to promote adhesion to substrates [8], and on its shell microstructure in adults [9]. As a prolific suspension feeder, *L. fortunei* has one of the highest reported clearance rates for suspension-feeding bivalves, including other invasive species such as *Dreissena polymorpha* (Pallas 1771), *Dreissena bugensis* (Andrusov 1897) and *C. fluminea*. This filtering capacity was analysed under laboratory conditions using cells from the alga *Chlorella vulgaris*. That also makes *L. fortunei* able to function as a bioindicator and sentinel of metal pollution and pollution monitoring, already observed in other bivalves such as the blue mussel *Mytilus edulis* (Linnaeus 1758) [10–12]. Indeed, this attribute has already been evaluated in studies involving the accumulation and dynamics of microplastics [13] and herbicides, such as glyphosate [14, 15].

Further understanding of the invasive mussels’ morphology can reveal their role in varying ecosystems and also provide insight into possible methods of population control in invaded areas [16]. A thorough morphological description of the *L. fortunei* anatomy has been reported by Morton [17]. *Limnoperna fortunei* has a single foot, two pairs of gills (ctenidia) and is gonochoric with external fertilization. Both juvenile and adult individuals have two valves surrounding the body, mainly composed of calcium carbonate [18] and its polymorphs aragonite and amorphous calcium carbonate [9]. The outermost part of the shell has a proteinaceous layer, known as periostracum. Adult shell length can reach 4.5 cm [17].

*Limnoperna fortunei* adult gills are flat, homorhabdic and filibranchiate [17], being in direct contact with the environment. In the presence of environmental contaminants chemicals, such as chlorothalonil, the bivalve gills are key in xenobiotics biotransformation, antioxidant response, innate immune response and osmoregulation [19]. Moreover, a giant virus belonging to the Marseilleviridae family was recently found in *L. fortunei* gills, as the morphophysiological structure of the gills favours microorganism bioaccumulation such as amoebas and viruses [20]. Bivalve gills are located in the mantle cavity [21]. After the post larvae stage, the gills are quite well formed in Mytilidae and Pectinidae, although they continue growing and developing until adulthood [22]. Each gill comprises two demibranchs, the outer and inner demibranchs, a double-lamellar macrostructure, namely ascending and descending lamellae [21]. The gill of the *L. fortunei* is type B(I) [17, 23], showing a W-shape in transverse sections, such as in *M. edulis* and other representatives of Mytilidae [22, 23]. The ventral margin of each demibranch has a deep groove, the marginal food groove [23]. Similar to *D. polymorpha* (Dreissenidae), the outer demibranch of *L. fortunei* is longer than the inner [17, 23]. This arrangement increases the efficiency of transfer of material from the marginal food grooves to the labial palps [17]. Each lamella comprises several parallel tubular filaments, the spaces between which form the interfilament channels. On the lateral surface of each individual filament there are ciliary bands, known as water-pumping cilia or lateral cilia (lc), responsible for the main water flow through the gills [17, 24]. Similar to other Mytiloida, there are frontal cilia (fc) at the frontal tract of the filaments, and laterofrontal cirri (lfc) located at the frontal margin of each filament frontal surface, in between the lc and fc. Each laterofrontal cirrus is a compound ciliary structure, and the action of the lfc facilitates particle capture [25, 26]. The fc transfer the captured particles towards the marginal food groove and then to the labial palps [17, 22, 24, 27–30]. Thereby, in addition to its respiratory function, this organ also fulfils the capturing and transportation of particles [17, 22].

Particle transportation on the gill filaments is mediated to a great extent by mucus [30–34]. The contact of captured particles to the fc of the gill filaments in *Ostrea edulis* (Linnaeus 1758) might cause the goblet cells to secrete mucus, trapping the particles within them [30]. Particles that require large amounts of mucus to cover them would be less likely to be ingested, while those demanding less mucus would be more likely to enter the labial palps [31]. In *M. edulis*, *Venerupis pullastra* (Montagu 1803) and *Cerastoderma edule* (Linnaeus 1758) an increase in mucus secretion was observed when particles were added to filtered sea water, and strings of mucus-particles could be observed [32]. Mucus mediating particle selection or rejection would be size dependent. High mass particles caused an instantaneous mucus discharge on the coarse frontal tracts of *Crassostrea virginia* (Gmelin 1791), in which the particles were entangled [33]. Mucus discharge would be triggered by a type of tactile stimulation. The smaller particles, on the other hand, would not cause such discharge to occur in the fine frontal tract [33]. A detailed mechanism of selection and rejection of mucus-particles strings by the labial palps in bivalves can be found in the works of Foster-Smith [32, 34] and Beninger and colleagues [35]. The mechanisms of particle capture were reviewed and discussed by Riisgård and Larsen [36] and the works of Ward and Shumway [37] and Rosa and colleagues [38] present great reviews of the present understanding of particle processing by suspension feeders.

The mechanism of particle processing and the further understanding of the physiological aspects of suspension-feeding bivalves greatly depend upon the knowledge of their morphology. *Limnoperna fortunei* morphology has been described in Morton [17]. Additionally, Paolucci and colleagues [39] reported the association between genetic variability and macro- and micro-structural morphology of *L. fortunei* populations across South America. However, few information about the ultrastructure of the golden mussel gills is available thus far. In this current work, for the first time, we characterized the ultrastructure of the gills epithelium of adult *L. fortunei.* These results will assist us to better understand the morphological aspects of the gills, which are vital for respiration and feeding.

## Results

### Gills microstructure

**Fig. 1:**
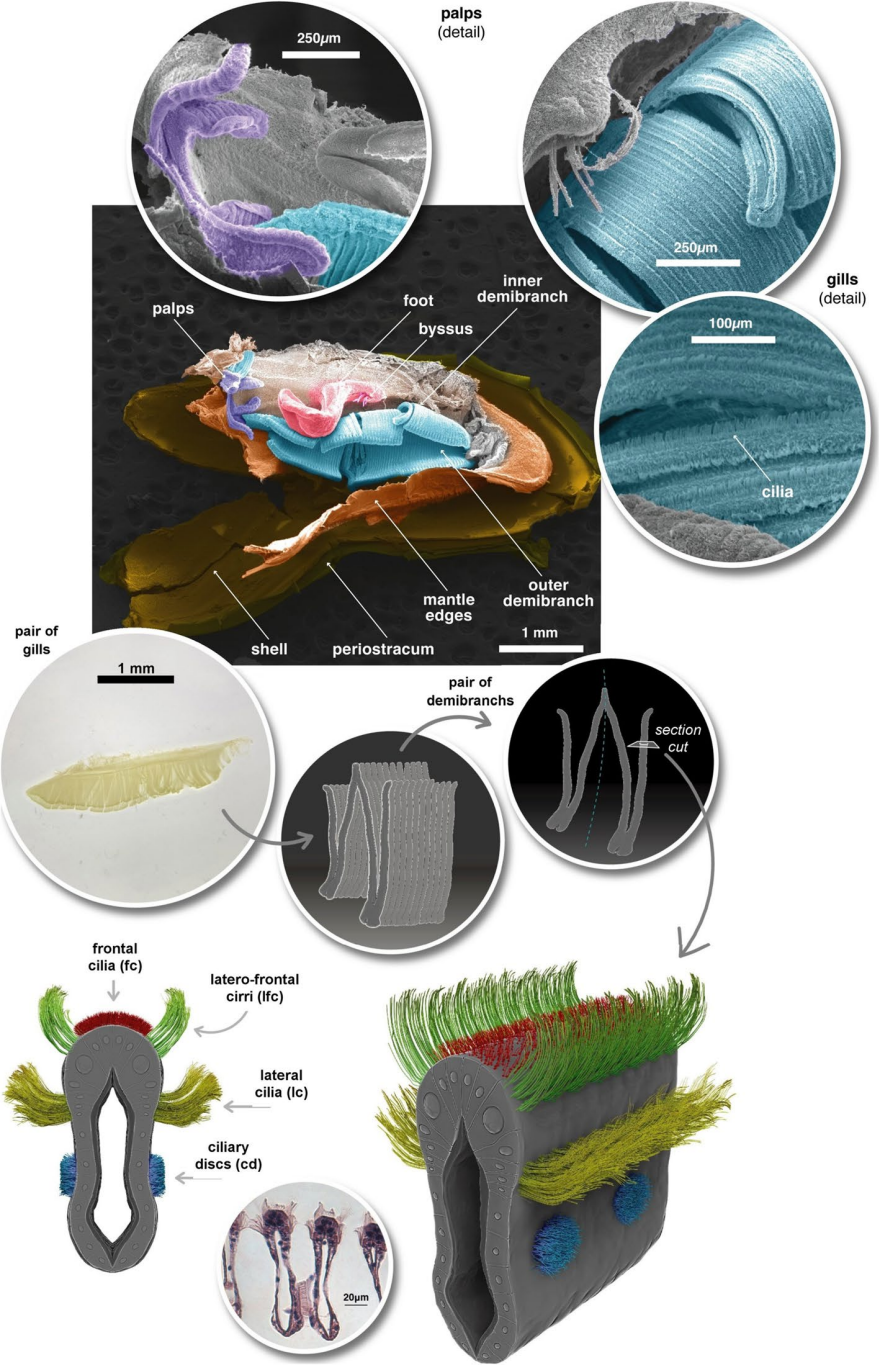
Digitally Coloured SEM images of *L. fortunei* with the left valve removed, showing one ciliated gill (inner and outer demibrach), the labial palps, the foot and byssus threads, and parts of the shell structure. The optical microscopy image of an isolated pair of gills, drawings of the W-shaped demibranchs, and of ciliated gill filament are shown at the bottom

**Fig. 2:**
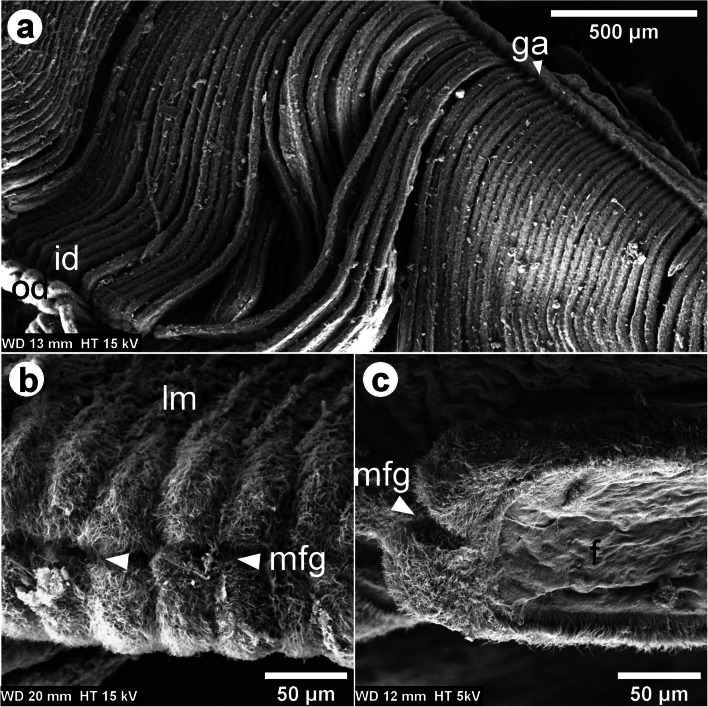
SEM images of the *L. fortunei* gills. **a** Dorsal view of the gill showing the lamellae, the inner- and outer- demibranch [id and od] joined by the gill axis [ga]. The od is behind and folded under the id. Its ventral margin appears at the lower left of the image. **b** Frontal view of one lamella [lm] showing its ventral aspect. **c** Longitudinal view of one filament demibranch [f] showing its lateral aspect. The marginal food groove [mfg] at the lamella’s edge is pointed out by the arrowheads in images (**b**,**c**)

The *L. fortunei* gills of adult individuals have a large surface area fitting the mantle cavity space. Each pair of gills has a leaf-like shape (Fig. 1) and is located at both sides of the viscera. Each gill comprises two demibranchs, known as inner- and outer- demibranchs, in a double lamellar structure joined by the gill axis (Fig. 2a). Each demibranch has nearly 75 filaments. Ventrally, the outer demibranch is longer than the inner, but it shortens laterally, close to the labial palps (Fig. 1).

**Fig. 3:**
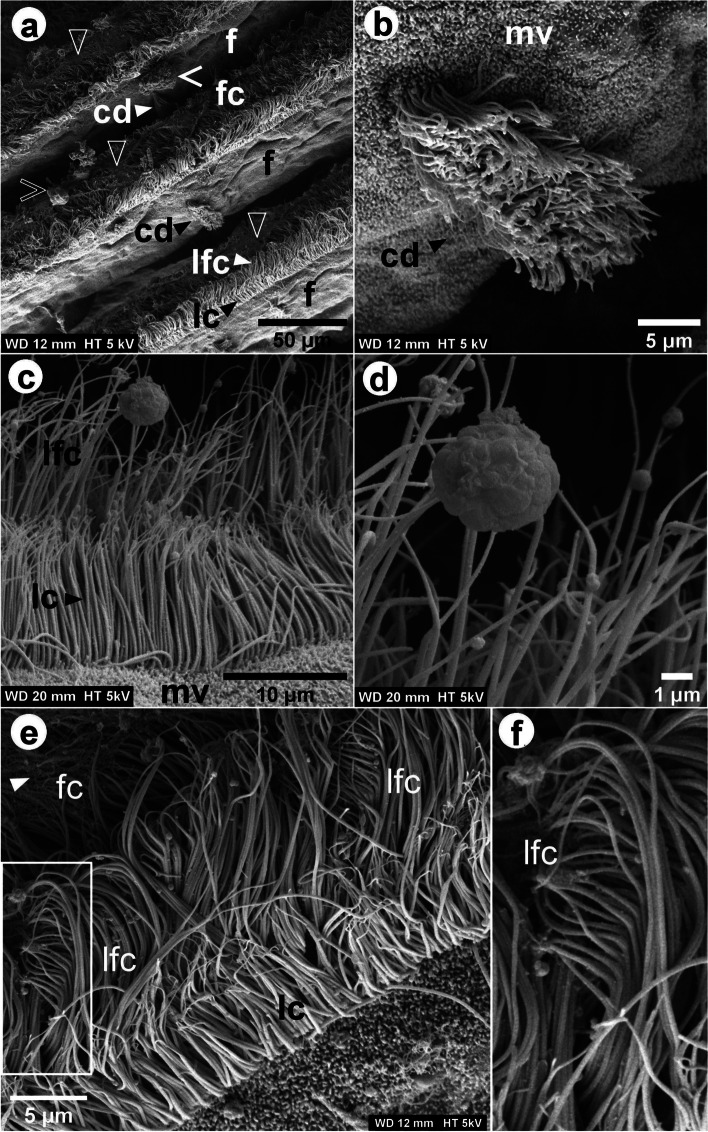
SEM images of the *L. fortunei* gills. **a** View in perspective of a set of filaments [f], showing ciliary discs [cd], lateral cilia [lc], laterofrontal cirri [lfc] and frontal cilia [fc]. The outlined arrowheads indicate likely mucus-strings on the frontal tract. The outlined and filled open arrowheads indicate larger particles on the frontal and lateral tract, respectively. Images (**b**) and (**c**, **d**) show in detail a cd, the lc and lfc, respectively. Microvilli [mv] are present below the lc and around the cd. Several spherical particles of about 200-300 nm and one of nearly 4-5 μm size were observed attached to the lfc. **e** View in perspective of one filament showing its lateral aspect and the detailed lfc at the top frontal margin. **f** Higher magnification image of the region marked with the rectangle in (**e**). Each individual cilia of the laterofrontal cirrus bent at the top, thus having a shape of a comb

**Fig. 4:**
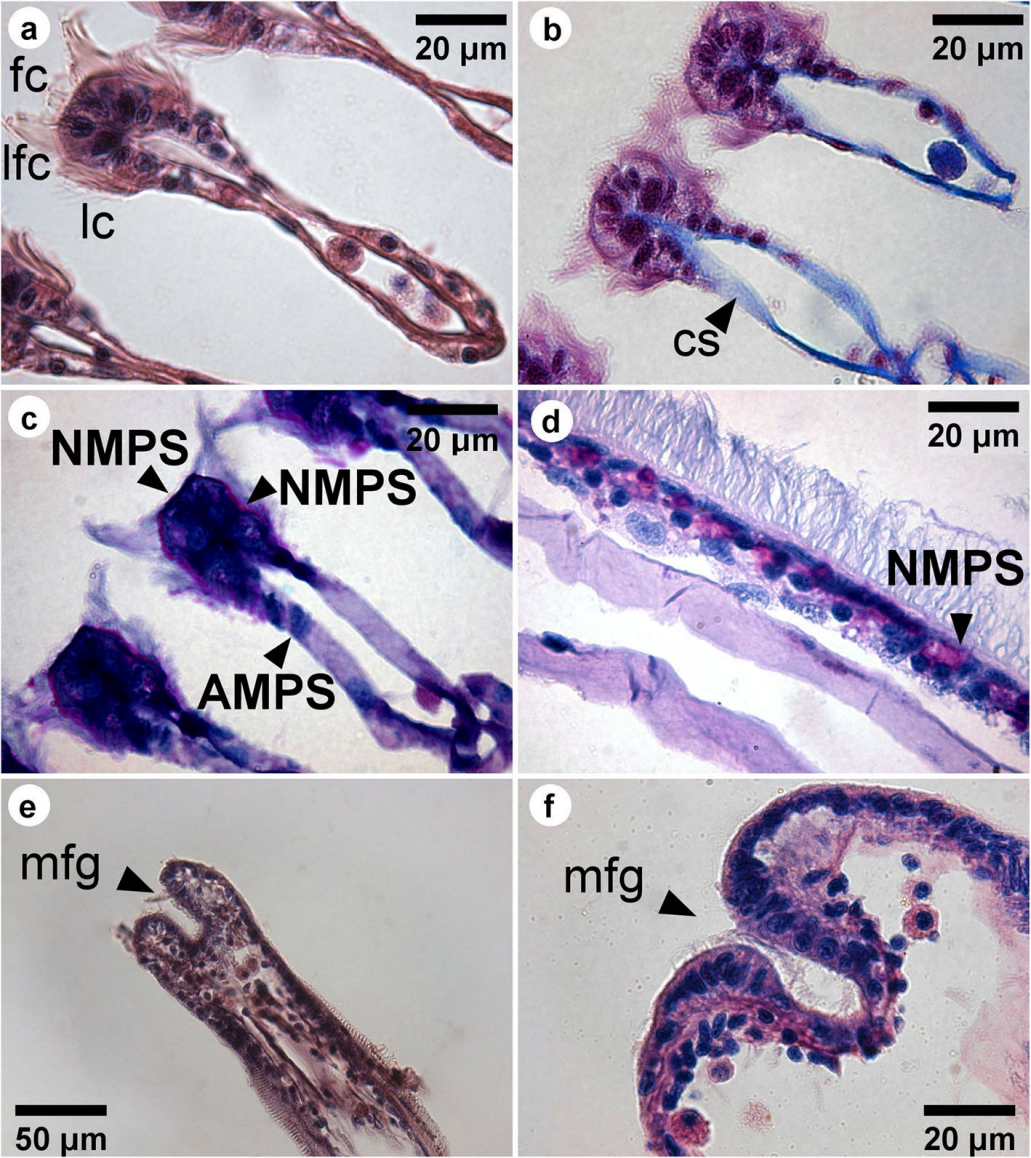
LM images showing the transverse (**a**-**c**) and coronal (**d**-**f**) sections of a gill filament of *L. fortunei*. Filaments stained with hematoxylin and eosin are shown in the images (**a**, **e**). The filaments stained with Alcian Blue/PAS shown in images (**c**, **d**, **f**), and stained with Masson’s trichrome in image (**b**). Neutral mucopolysaccharides (NMPS) are coloured in pink and acid mucopolysaccharides (AMPS) are coloured in blue. Lateral cilia [lc], laterofrontal cirri [lfc], frontal cilia [fc], marginal food groove [mfg], and collagenous structure [cs] are indicated

**Fig. 5:**
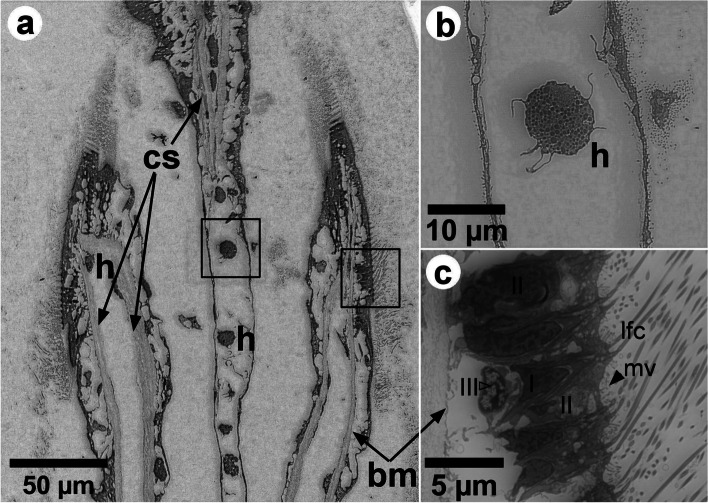
**a** Backscattered electron SEM images (with inverse contrast) of a thin section longitudinal to the gill filament close to the marginal food groove (at the top, not visible). The collagenous structure [cs] supporting the hemolymph vessel of the epithelium, the smooth basal membrane [bm], and hemocytes [h] are shown. **b**, **c** Higher magnification images of the squares shown in (**a**). A lobed hemocyte in the central hemolymph shown in (**b**). Large vacuoles, cs and bm are shown in (**c**). Three types of cells are indicated, types I and II with a dark nuclei, and the type III with a bright nucleus. The filled arrowheads indicate the microvilli on the apex of cells of type II. The lfc that arise from the cells of type I are also shown

**Fig. 6:**
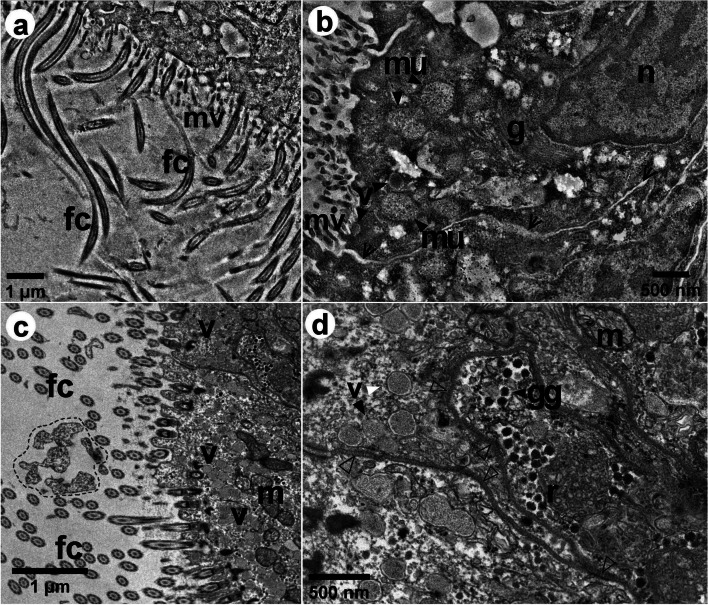
Bright-field TEM images of the epithelial cells of *L. fortunei*, showing coronal sections of a gill filament. In image (**a**), many frontal cilia [fc] are observed longitudinal to the gill filament, and in image (**c**) the fc are observed in cross-section. **b**, **d** Epithelial cells at higher magnification, displaying the nucleus [n], mitochondria [m], Golgi complex [g], vesicles [v], smooth reticulum [r], and glycogen granules [gg]. The region highlighted in image (**c**) shows possibly mucus in between the fc. The filled arrowheads in images (**b**, **d**) point out some vesicles, mitochondria, and glycogen granules. The open arrowheads in (**b**) indicate apparent non-junctional regions of the cell membranes with a slightly increased space. The outlined arrowheads in (**d**) indicate the septate junctions

**Fig. 7:**
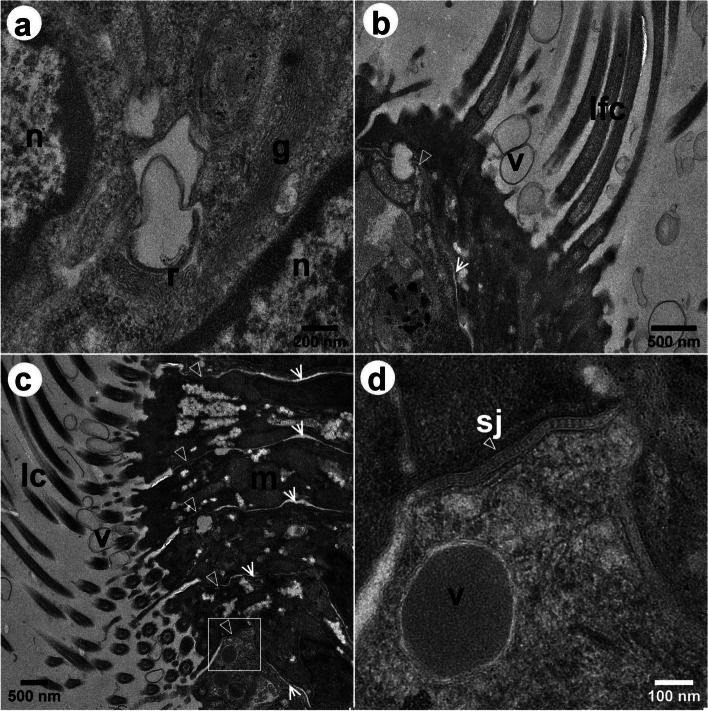
Bright-field TEM images of the epithelial cells of *L. fortunei*, showing transversal sections of the gill filament. **a** Epithelial cells of the frontal tract, displaying the nucleus [n], Golgi complex [g] and smooth reticulum [r]. **b** Cells at the margin of the frontal tract showing the laterofrontal cirri [lfc] and spherical vesicles [v] above the epithelium. **c** Cells of the lateral tract that bears the lateral cilia [lc]. Spherical vesicles are also present in between the lc. **d** Higher magnification image of the square in **c** showing vesicle possibly containing mucus, and the septate junction in detail [sj]. The outlined arrowheads in images (**b**-**d**) indicate septate junctions. The open arrowheads point out non-junctional regions of the cell membranes with increased space

At the margins of the frontal surface of each filament, different ciliary projections were observed: lc and lfc, and, on the frontal surface, fc (Figs. 1, 3, 4 and 5 and Additional file 1). Each laterofrontal cirrus has approximately 18-28 pairs of cilia (see Additional file 1). Ciliary discs (cd) were observed at the lateral surface of the filaments (Fig. 3), measuring approximately 16 × 10 μm and cross-connecting individual filaments. TEM images showed that lc, lfc, and fc have the type 9+2 axoneme microtubule-based cytoskeleton (Additional files 1, 2 and 3, Figs. 6 and 7). Pro-laterofrontal cirri (p-lfc) could not be observed between the lfc and fc in the SEM and TEM images. Several adhering particles (<15 μm) could be observed on the frontal surface of the filaments (Fig. 3). A larger particle (nearly 20 μm) could be observed at the lateral tract (Fig. 3) and several smaller particles were observed attached to the lc and lfc (Fig. 3c-d). The smallest particles (200-300 nm) were found to be spherical vesicles and clearly seen on TEM images (Fig. 7b-c, Additional files 1 and 2). Particles of almost 4-5 μm are probably the size of algae cells.

### Gills ultrastructure

Light microscopy (LM) images show transverse and longitudinal sections of gill filaments (Fig. 4). The lc, lfc and fc can be clearly observed in the transverse section (Fig. 4a-b). Based on the combined alcian blue and periodic acid Schiff (AB-PAS) staining, we could observe the presence of different types of mucocytes in the gills filaments sections. Acid and neutral mucopolysaccharides were also observed. Larger amounts of neutral mucopollysaccharides (NMPS) were found at the apex of the frontal surface (Fig. 4c-d - stained in pink), while acid mucopollysaccharides (AMPS) were found, in small numbers, spread out in the whole filament (Fig. 4c-d - stained in blue).

TEM image of the transverse section of one filament showed many ciliated epithelial cells (see Additional file 6). Backscattered electron (BSE) SEM images of the longitudinal section of filaments are shown in Fig. 5, with an inverse contrast resembling TEM images. At the dorsal part of the filament epithelium, the basal membrane is smooth (Fig. 5 and Additional file 2). It is supported by a collagenous structure, which also surrounds the hemolymph vessel (Fig. 5). Close to the marginal food groove, a V-shaped collagenous structure could be observed (Fig. 5a). It is apparent that the central hemolymph vessel has no collagenous supporting structure, apart from the marginal food groove. Hemocytes were observed in the central hemolymph (Fig. 5a-b), while more elongated hemocytes could be observed in the region below the basal epithelium (Fig. 5a-c). Three types of cells were observed, Two of them (cells I and II) located at the apical epithelium and the other (cell III) at the basal region (Fig. 5c and Additional file 7). The cell I narrows at the apex of the epithelium and has an elongated dark nucleus that occupies a large volume in the cell and contains more dispersed heterochromatin. Each laterofrontal cirrus arises from a single cell I, as can be seen in the 3D model (see Additional file 7). In between these cells, we could observe the cell II, which has a goblet shape also with an elongated dark nucleus, but with less dispersed heterochromatin. Cell II enlarges at the apex of the epithelium and possesses microvilli, 880 ± 150 nm long (Fig. 5c Additional file 7). The microvilli were also observed on the surface of the lateral tract of the filaments, below the lc around the cd (Fig. 3b-c). Numerous mucins were observed in the cells II (Additional file 8). The cells III have a lobed bright nucleus and were mostly found present at the basal epithelium (Figs. 4d and 5c). Vacuoles occupy a large volume of epithelium and interconnect the basal membrane to the apical region (Fig. 5c).

Gill epithelial cells are shown in Figs. 6 and 7. In Fig. 6a, some fc lay longitudinally to the demibranch filament, suggesting that the fc are stiffer in that portion of the filament, as also observed in Fig. 3c and Additional file 5. In Fig. 6c, the fc were observed in cross-section, which means most of them are bent. Many mitochondria could be observed, mainly in the region close to the insertion site of gill cilia (Fig. 6c-d), while the smooth endoplasmic reticulum could be found in the apical region (Fig. 6d). Surrounding the smooth reticulum, we could also observe several nearly 70 nm electron dense glycogen granules (Fig. 6d). Cell junctions could clearly be seen in the portion of the epithelium observed in TEM images (Figs. 6 and 7) and several septate junctions were observed adjacent to the region with glycogen granules and smooth endoplasmic reticulum (Figs. 6d and 7b-d). In addition, numerous vesicles (201 ± 29 nm), possibly containing mucus, could also be observed in this region (Figs. 6b-c and 7c-d).

## Discussion

In this work, we have characterized the ultrastructure of the mucociliary epithelium of *L. fortunei* gills in order to describe the cellular traits of this important biological structure, mainly responsible for feeding and respiration. The gills of suspension feeders are in direct contact with the environment and understanding their morphology is fundamental to the establishment of new strategies to manage this invasive species in the environment.

Adult gills size from the specimens used in the present work did not significantly differ from the population of *L. fortunei* studied by Paolucci and colleagues [39]. In this work, we could observe a slightly longer mean cilia length of fc of the Volta Grande (VG) specimen, and a slightly lower mean of filament width for the Paranaíba river (PR) one, both compared with *L. fortunei* populations of South America [39]. Morphometric differences found for both VG and PR specimens might be due to different environmental conditions in which they had grown and adapted to.

Classically, the two main functions of the gills in bivalves are feeding and respiration. The thin structure of the gill epithelium may allow the exchange of gases such as oxygen and carbon dioxide by passive diffusion, in response to partial gas pressures. Also, it allows ion exchange between the external environment and the hemolymphatic vessels [40]. The observed microvilli at the apical pole of goblet cells of *L. fortunei* are now evidence that gills might also present a trophic function. The outermost gill epithelium might have a large surface area due to the microvilli, which may assist the direct uptake of dissolved or particulate organic matter. This trait was also suggested for the bivalve *Placopecten magellanicus* (Gmelin 1791) [41, 42]. Furthermore, these cells present vacuoles interconnecting the apical region to the basal membrane of the epithelium, which suggests that transport and diffusion of nutrients might be occurring [42]. We also found endoplasmic reticulum and Golgi complex, organelles that produce and excrete mucus in the goblet cells. These cells look similar to the ones found in *M. edulis* [43] and *P. magellanicus* [42]. As described for the Brazilian endemic bivalve *Diplodon expansus* (Küster 1856) [44], the production of mucus in this apical region of the gills might be associated with lubrication, in order to reduce the frictional resistance in water flow along the epithelium. In *D. expansus*, the mucus layer is highly viscous and difficult to hydrate, ensuring the efficiency of the mucus as a lubricant. The mucus associated with the ciliary tracts might change the local fluid mechanical properties and, in fact, only a small amount of mucus is needed for the viscosity of the medium transport [45]. It is unlikely that captured particles can be kept in this confined local current produced by the cilia beating without the intervention of mucus in mytiloids homorhabdic gills [36, 46]. Particles covered by intermediate-viscosity mucus are transported close to the frontal gill epithelium in *M. edulis*, in such enclosed space [32, 44]. Our own results are evidence of that in *L. fortunei*. Numerous vesicles, mucus and likely mucus strings were observed in the fc tract of *L. fortunei*. The LM images of sections stained with AB-PAS showed mixed-secreted (neutral and acid) mucopolysaccharide in the gill filaments, with NMPS being abundant on the frontal surface of the filaments. This result corroborates other studies in *M. edulis* [45] and in the oyster *Crassostrea gigas* (Thunberg 1793) [47]. The particles bound to NMPS or mixed (acid + neutral) muccopolysaccharides are transferred to the marginal groove by the frontal cilia and then transported to the labial palps, where they are sorted and either ingested or rejected as pseudofeces.

The mechanisms of particle capture and their transport in suspension-feeding molluscs are almost exclusively ciliary dependent. The lc are responsible for pumping water through the gill interfilament channels towards the suprabranchial cavity [24, 27, 42]. One of the functions of the lfc in Mytilidae and Pectinidae is ascribed to the particle capture [13, 22, 24, 27], which is accomplished by the lfc [27, 28], or through currents produced as the lfc beat against the main water current [24]. Captured particles are then transported towards the marginal food groove by the action of fc, which seems to be autonomous mechanical processes. However, *in vivo* endoscopic observations in many bivalves have shown that the transportation of particles depends either on mucociliary and hydrodynamic mechanisms [25, 26]. Particle rejection or ingestion, on the other hand, is based on physicochemical interactions that can be sensed in the labial palps [13, 42].

Particle selection mechanism is not fully resolved. Some works show that it depends on particle characteristics such as size, shape and surface properties, which affect their ingestion or rejection [37, 38]. Rejected particles are bound to cohesive mucus, deposited in specific sites of the mantle and then transported to the cilia, for their expulsion as pseudofeces [25, 48]. Additionally, the mucus covering feeding organs might mediate particle selection [13, 33, 49]. For *P. magellanicus*, it has been shown that reduced mucus-particles viscosity are more likely to be ingested, while high viscosity mucus-particles are more likely rejected (AMPS) [49]. Our own results show evidence that the mucus produced and excreted in the gills epithelium also plays a role in the mechanism of particle capture itself.

Our data suggested that the mucus in *L*. *fortunei* gill filaments might be correlated to the fc beating. A larger number of mucus-containing vesicles in the epithelium cells were observed when the fc were bent and, conversely, less mucus-containing vesicles were observed when fc were stiffer, being longitudinal to the gill filament. Such correlation is rather difficult to ensure based on images of TEM sections only and it would need to be investigated by volume electron microscopy. TEM images suggested that the mucus are packed and sent to the apical region. Indeed, we observed mucus and spherical vesicles above the epithelial cells in between the fc and lfc and also mucus covering a food particle. This might be an ongoing process, in which the mucus carried by vesicles towards the gill cilia might be available to interact with the surface of an upcoming particle that will be captured and further transported to the labial palps to be physicochemically sensed and discriminated [13, 42], and then rejected or selected by the feeding organs [13]. The mucus produced by epithelial cells is modified by the Golgi complex, near the nucleus, playing a key role in sorting newly synthesized and recycled molecules towards their final destinations [50], the apical region of the epithelium. TEM images also showed mitochondria with extensive lamellar cristae, arranged in parallel juxtaposed sheets that occupy most of the organelle volume, which is common of high energy-demanding tissues [51] such as the gill ciliary epithelium [52]. The smooth reticulum was also present and among several cellular functions, it participates in glycogen metabolism [53]. The glycogen granules are important components for the bivalve metabolism [54]. Indeed, several septate junctions were observed interconnecting the cell rich in mucus vesicles to the cell where glycogen granules and smooth reticulum were observed. The presence of the septate junctions in this portion of the epithelium is evidence of the active intercellular communication and transport of molecules between them [55, 56]. This energetic apparatus is related to the morpho-functional structure of the ciliary epithelium and its analysis allowed us inferring the correlation between the mucus in gill filaments to the beat of the cilia. Such correlation is quite hard to confirm by single TEM sections, though. This would require a 3D reconstruction of the ciliary epithelium at high spatial resolution. This will be further investigated by volume electron microscopy techniques, to better understand such dynamical processes as this.

## Conclusions

Understanding the *L. fortunei* morphology is the first step towards the establishment of strategies to control this invasive species and, to the authors’ knowledge, this is the first time high-resolution ultrastructure of *L. fortunei* gills epithelium have been reported. Our data showed the microstructure of the gill filaments and cilia in high spatial resolution and also evidence of the production and release of mucus and spherical vesicles in ciliary cells. This might have implications to the process of selection and discrimination of particles to be ingested or rejected by mussels.

## Methods

### Gills preparation

Specimens of adult *L. fortunei* were collected in February 2019 in the fish farming reservoir of the Volta Grande (VG specimen), on the border between the states of Minas Gerais and São Paulo, Brazil (20°01’54.0"S, 48°13’10.0"W), where measured water parameters were 29.2 ºC, pH 7.5, dissolved oxygen 3.2 mg L^−1^, and turbidity 0.1 NTU. Specimens were packed in cloth bags (to decrease overlapping individuals) and during transport, they were submerged in water at constant aeration. In the laboratory at the *Centro de Bioengenharia de Espécies Invasoras de Hidrelétricas* (CBEIH), nearly 200 animals were acclimated for 3 weeks in an aquarium with 36 L capacity containing artesian water well, pH 7.7, dissolved oxygen 6.8 mg L^−1^ and turbidity 1.68 NTU, at constant aeration and temperature of 18 to 20 ºC to minimize stress. After acclimation, the molluscs were kept under the same conditions and temperature of 22 ± 1 ºC.

### Light microscopy

Adult *L. fortunei* specimens (n=10) were taken out of the aquarium and immersed in Bouin’s fixative for 24 h. After this, the gills were dissected, dehydrated in a progressive series of ethanol, cleared in xylene and then embedded in paraffin. Longitudinal and transverse histological sections of the gills were cut with 5 μm thickness, using a MRS 3500 Microtome. Sections were dewaxed in xylene, hydrated in graded ethanol and stained accordingly: to determine the general structure of gills filaments, sections were stained with hematoxylin-eosin [57]; to demonstrate the existence of polysaccharides, periodic acid Schiff (PAS), combined with alcian blue (AB), at pH 2.5, were used [58], which allowed us differentiating between neutral (stained pink) and acid polysaccharides (stained blue). In addition, the Masson’s trichrome method [59] was used to allow the identification of structures that had connective tissue.

### Electron microscopy

For the purpose of this work, an adult specimen, with a shell length of approximately 1.5 cm long, was taken out of the aquarium so its valves could be kept partially open with the help of a short piece of metal (1 mm diameter). Next, the specimen was rapidly submerged in a 1.5 mL Eppendorf® tube, filled with modified Karnovsky fixative solution (2% paraformaldehyde and 2.5% glutaraldehyde), and incubated for 3 days. The valves were then carefully opened and the gills dissected using forceps, to further process for scanning electron microscopy (SEM) and transmission electron microscopy (TEM). Dissected gills were placed into 1.5 mL Eppendorf® tubes containing phosphate buffer solution (PBS). In this present work, we also used unreported TEM and SEM data of the gills from another pristine adult specimen that was collected in Paranaíba river (PR), downstream the confluence with Barreiro’s river, near the municipality of Paranaíba (Mato Grosso do Sul, Brazil). Sample preparation details about this latter specimen can be found in Andrade et al. [8]. The two specimens used in the work were named after the place they were collected, as VG and PR specimens.

### Scanning electron microscopy (SEM)

Immediately before the secondary fixation, PBS excess volume was first removed and replaced by an appropriate volume of a fresh PBS and incubated for 10 min. This washing process was performed three times. PBS was then replaced by an appropriate volume of 1% osmium tetroxide (OsO_4_) in PBS (pH 7.3 ± 0.1) in the fume hood and incubated for 1 h in darkness and room temperature (RT). The sample was washed with PBS and incubated for 10 min, three times. Excess PBS was removed and replaced by 1% tannic acid (C_76_H_52_O_46_) solution and incubated for 20 min at RT. After washing with PBS three times, the solution was replaced by 1% OsO_4_ solution and incubated for 1 h in darkness and RT. The samples were then washed in distilled water three times and dehydrated in a sequence of alcohol solutions (35%, 50%, 70%, 85%, 95% and 100%), 10 min each. The last step with absolute alcohol was performed twice. The sample was critical point dried with CO_2_ (using a Leica EM CPD 030), placed on an aluminium SEM stub with a carbon tape and finally coated with gold nanoparticles (5 nm thickness) in a sputter coater (Bal-tec MED 020). The VG sample was analysed in a field emission scanning electron microscope (FEI Quanta 200), operated at 5 kV and 15 kV.

### Transmission electron microscopy (TEM)

For TEM analysis, samples were firstly washed three times in PBS, for 10 min each time. PBS was replaced by 2% OsO_4_ in PBS (pH 7.3 ± 0.1) and incubated for 2 h in darkness at RT. Samples were then washed with deionized water four times, for 10 min each time. Distilled water was then replaced by 2% uranyl acetate (C_4_H_8_O_6_U) solution and incubated overnight at 6ºC in darkness. Samples were again washed with deionized water, three times, for 5 min each, followed by dehydration in a sequence of alcohol solutions (35%, 50%, 70%, 85%, 95% and 100%). Absolute alcohol was replaced by acetone and incubated for 20 min. Acetone was then replaced by Epon^TM^ resin in three steps, using different dilutions of acetone in resin (2:1, 1:1, and 1:2). In each of these three steps, the tube was enclosed and homogeneously agitated (using a Norte Científica NH2200) for at least 2 h. After drying the excess acetone/resin with a filter paper, samples were transferred to a new polypropylene tube containing Epon^TM^ resin prepared with DMP-3 (Sigma Aldrich) and incubated at 40ºC for 1 h. Samples were carefully embedded to obtain longitudinal sections of the lateral surface of the gills’ filament. Ultrathin Sect. (60 nm) were obtained using an ultramicrotome (Leica EM UC6) and a diamond knife Ultra 45º (Diatome), with the sections being transferred to C-film Cu-TEM grids. Series of ultrathin Sect. (100 nm) array was also obtained and deposited in a clean silicon wafer. The surface of the silicon wafer was previously glow discharged so to become more hydrophilic. Sections were post-stained using a 2% uranyl acetate and lead citrate. TEM analysis was performed in a thermionic W-filament transmission electron microscope (FEI Tecnai Spirit G2-12 BioTwin), operated at 120 kV. SEM and TEM samples preparations and their analysis were performed at the Center of Microscopy at the Universidade Federal de Minas Gerais (UFMG).

### Structural analysis

The micro-morphological analysis of the *L. fortunei* gills structure was performed using SEM images. Electron microscopy of thin sections was used to describe the ultrastructural morphology of the ciliary gill epithelium. When suitable, measurements of the gill filaments were performed using transmitted light microscopy images (Additional file 4), taken in a Leica DM4500 microscope, with the same plastic embedded blocks for TEM preparation. Filament width, interfilament space, and filament linear density (number of filaments per linear distance) were obtained from SEM images. Data are shown in the Additional file 9. Cilia length was obtained in SEM images and the cilia diameter and linear density (number of cilia onto the filament over the linear distance along the filament) using the TEM images. Measurements were performed using the free software FIJI / ImageJ v. 1.52p (Wayne Rasband - National Institutes of Health, USA) and the DigitalMicrograph® v. 3.41.2916.1 (Gatan, Inc).

## Supplementary Information

**Additional file 1. **TEM bright field imageshowing coronal section of a filament of*L. fortunei*’s gill. Thelaterofrontal cirri [lfc] are shown in cross-section view.

**Additional file 2. **Montage of TEM bright fieldimages showing longitudinal view of the whole gill epithelium*.* Legend: cs – collagenous supportingstructure, n – nucleus, vc – vacuoles, mu – mucin, mv – microvilli, lfc –laterofrontal cirrus, fc – frontal cilia, v – vesicle.

**Additional file 3. **TEM bright field images showing the detail microtubulecytoskeleton of laterofrontal cirri in cross-section (c) and longitudinal (b)views.

**Additional file 4. **Transmitted light microscopy of the plastic embeddedblock of the mussel gill in Volta Grande (VR) specimen.

**Additional file 5. **SEM images of the frontal and lateral tract of gillfilament. The white arrowhead points a 4-5 µm particle on the frontal tract.Legend: ms – mucus string, fc – frontal cilia, lfc – laterofrontal cirri, cd –ciliary dics.

**Additional file 6. **Montage of bright-field TEM images of a thin sectiontransversally to a gill filament of *L.fortunei,* showing its frontal portion. Lateral cilia [lc], laterofrontalcirri [lfc], and frontal cilia [fc] are observed in cross-section view.Spherical vesicles [v], vacuoles [vc], the collagenous structure [cs] of the hemolymph,and a hemocytes [h] are indicated.

**Additional file 7. **Three dimensional reconstruction of a portion of the *L. fortunei* gill epithelium showingthree different types of cells. Cell I show a colored green nucleus and bearthe laterofrontal cirri (in ligh gray). Cell II shows a colored orange nucleus.It possesses microvilli (in red) and is positioned in between cell I. Cell IIIshows a colored dark green and is located at basal region of the epithelium.Spherical vesicles are located inside cell II (in yellow) and outside (inbeige). The reconstruction was carried out by serial-sectioning-array BSE-SEMimages and using the Free-D software: Andrey P., Maurin Y. (2005). J Neurosci Methods.145(1-2):233-44. doi:10.1016/j.jneumeth.2005.01.006.

**Additional file 8. **Bright-field TEM images showing coronal thin sectionsof the gill epithelium of *L. fortunei*.The laterofrontal cirri [lfc] are shown in cross-section view. Mucins [mu] andvacuoles [vc] are observed in the cells that possess microvilli [mv]. TSphericalvesicles [v] are present close to the lfc and septate junctions [sj] and nuclei[n] are indicated.

**Additional file 9. **Measurements of the gills structure variables (mean ±standard deviation) of the Volta Grade (VG) and Paranaíba River (PR) specimens.Errors were estimated from measurements performed in different parts of theimage.

## Data Availability

The datasets used and analysed during the current study are available from the corresponding author on reasonable request.

## Abbreviations

3D: Three dimensional
AB-PAS: Alcian blue and periodic acid Schiff
AMPS: Acid mucopollysaccharides
BSE: Backscattered electron
CBEIH: Centro de Bioengenharia de Espécies Invasoras de Hidrelétricas
cd: Ciliary discs
fc: Frontal cilia
lc: Lateral cilia
lfc: Laterofrontal cirri
LM: Light Microscopy
NMPS: Neutral mucopollysaccharides
PBS: Phosphate buffer solution
p-lfc: Pro-laterofrontal cirri
PR: Paranaíba river
SEM: Scanning Electron Microscopy
TEM: Transmission Electron Microscopy
VG: Volta Grande

## Declarations

Our experiments were performed using adult *L. fortunei* specimens (which are molluscs of the class *Bivalvia*). The collection, transport and maintenance of the molluscs in the laboratory have been authorized by the environmental agency ICMBio, linked to the Ministry of the Environment under code 72222-2 (and can be accessed in https://drive.google.com/file/d/1SVsAlcTtghxozTmYpyVu9JhEvKX0eMQx/view?usp=sharing).
