# Supplementary figures and images for: Ultrastructure of the gill ciliary epithelium of Limnoperna fortunei (Dunker 1857), the invasive golden mussel
Source: BMC Zool. 2022 Jan 17;7:6. doi: 10.1186/s40850-022-00107-y (PMC10127303; doi:10.1186/s40850-022-00107-y)

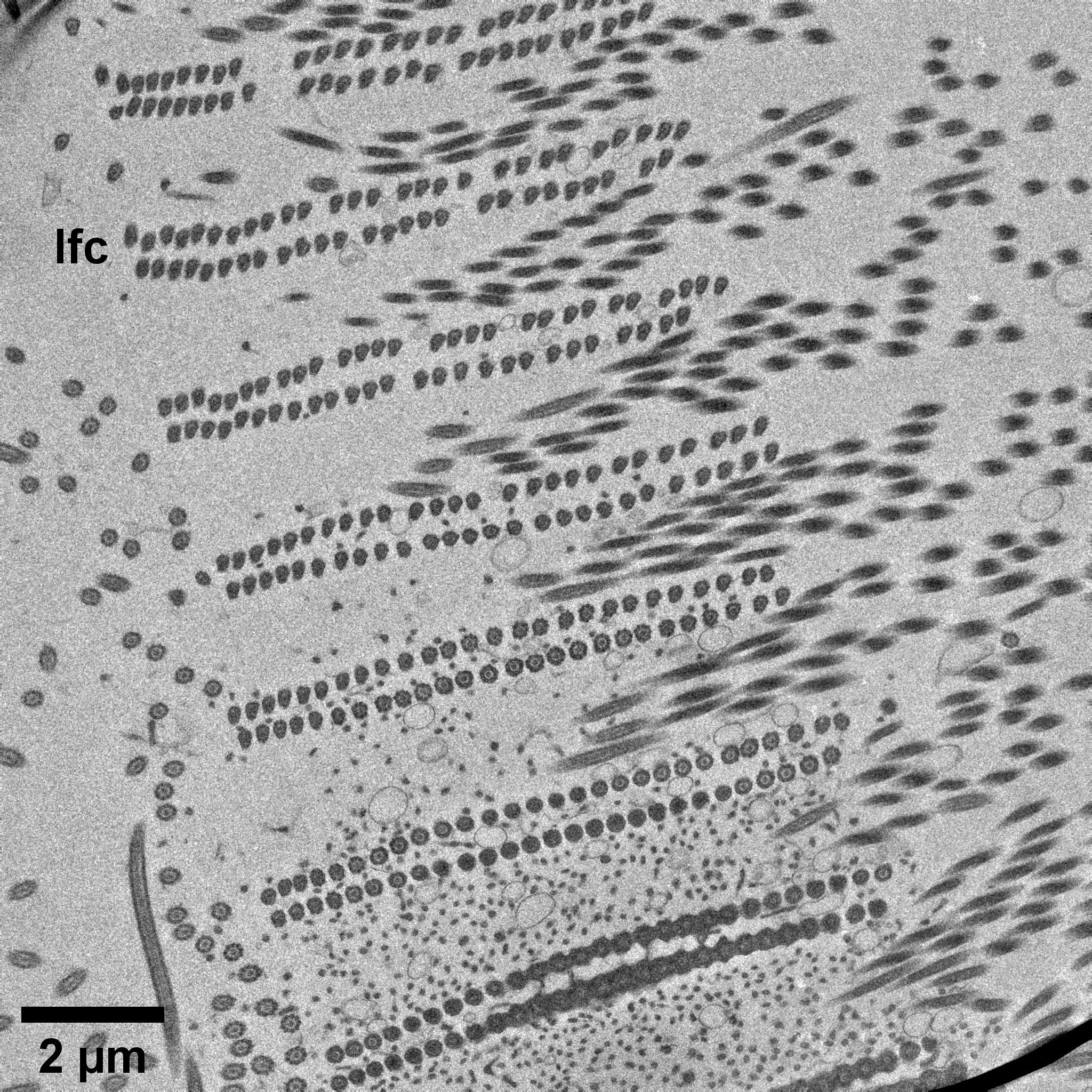

Supplement: Supplementary file 1 — Additional file 1. TEM bright field imageshowing coronal section of a filament ofL. fortunei’s gill. Thelaterofrontal cirri [lfc] are shown in cross-section view. [file 40850_2022_107_MOESM1_ESM.tif]

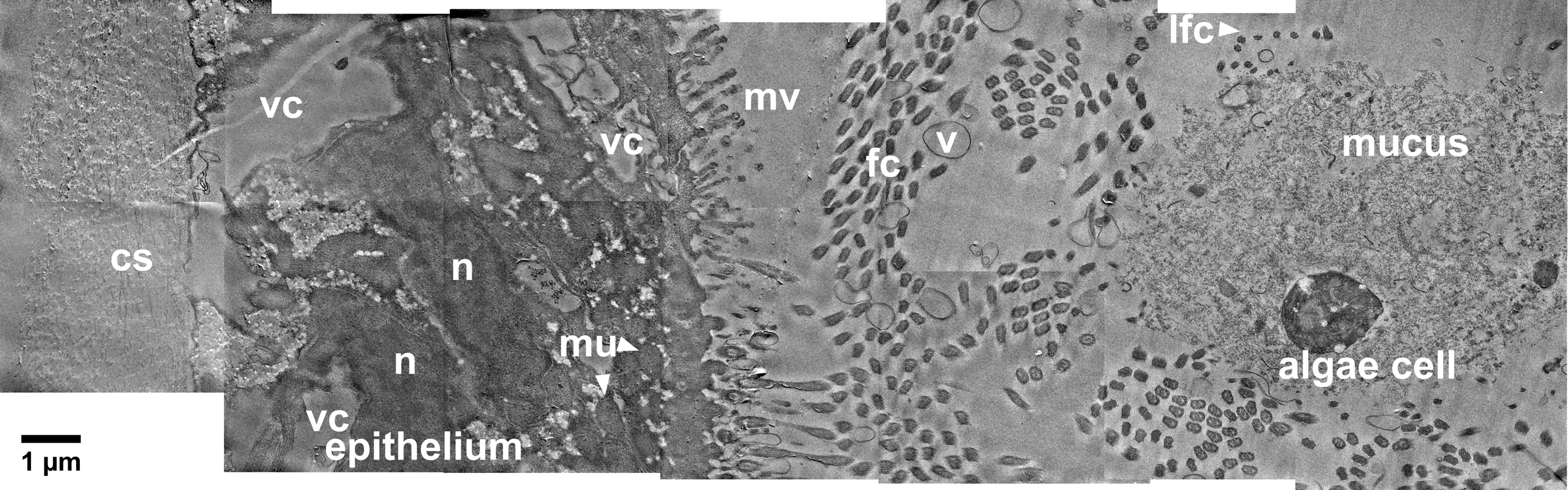

Supplement: Supplementary file 2 — Additional file 2. Montage of TEM bright fieldimages showing longitudinal view of the whole gill epithelium. Legend: cs – collagenous supportingstructure, n – nucleus, vc – vacuoles, mu – mucin, mv – microvilli, lfc –laterofrontal cirrus, fc – frontal cilia, v – vesicle. [file 40850_2022_107_MOESM2_ESM.tif]

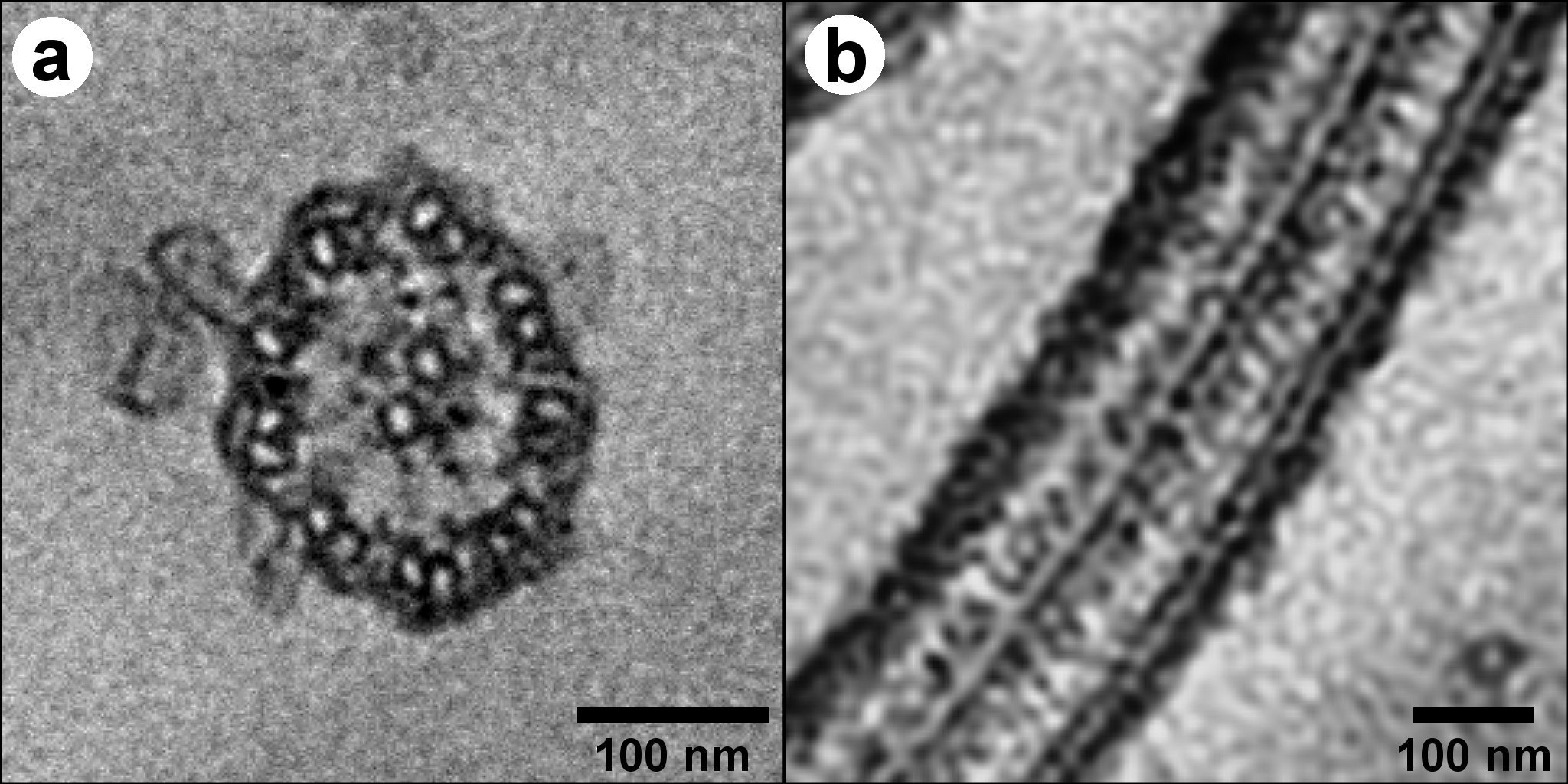

Supplement: Supplementary file 3 — Additional file 3. TEM bright field images showing the detail microtubulecytoskeleton of laterofrontal cirri in cross-section (c) and longitudinal (b)views. [file 40850_2022_107_MOESM3_ESM.tif]

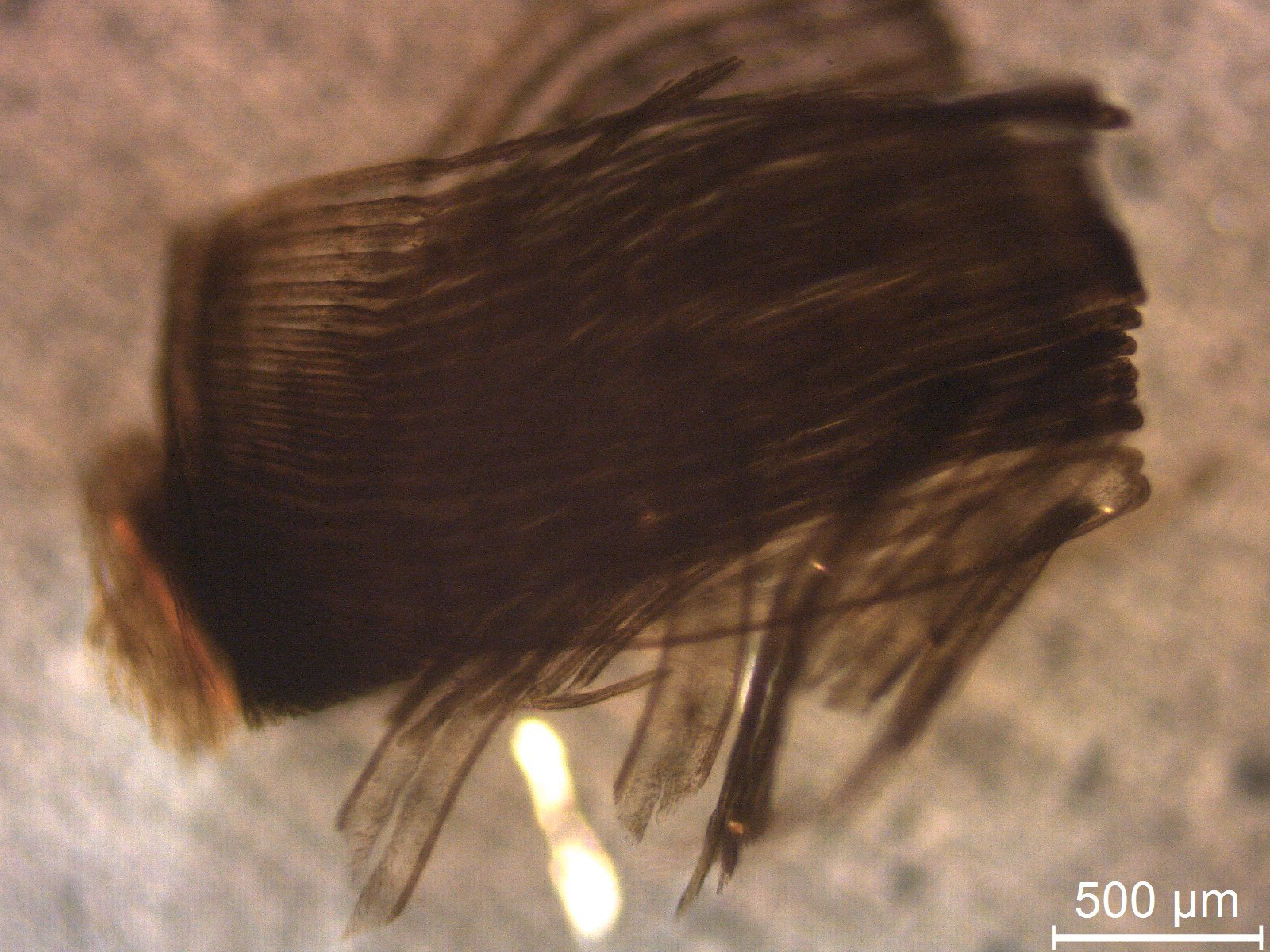

Supplement: Supplementary file 4 — Additional file 4. Transmitted light microscopy of the plastic embeddedblock of the mussel gill in Volta Grande (VR) specimen. [file 40850_2022_107_MOESM4_ESM.tif]

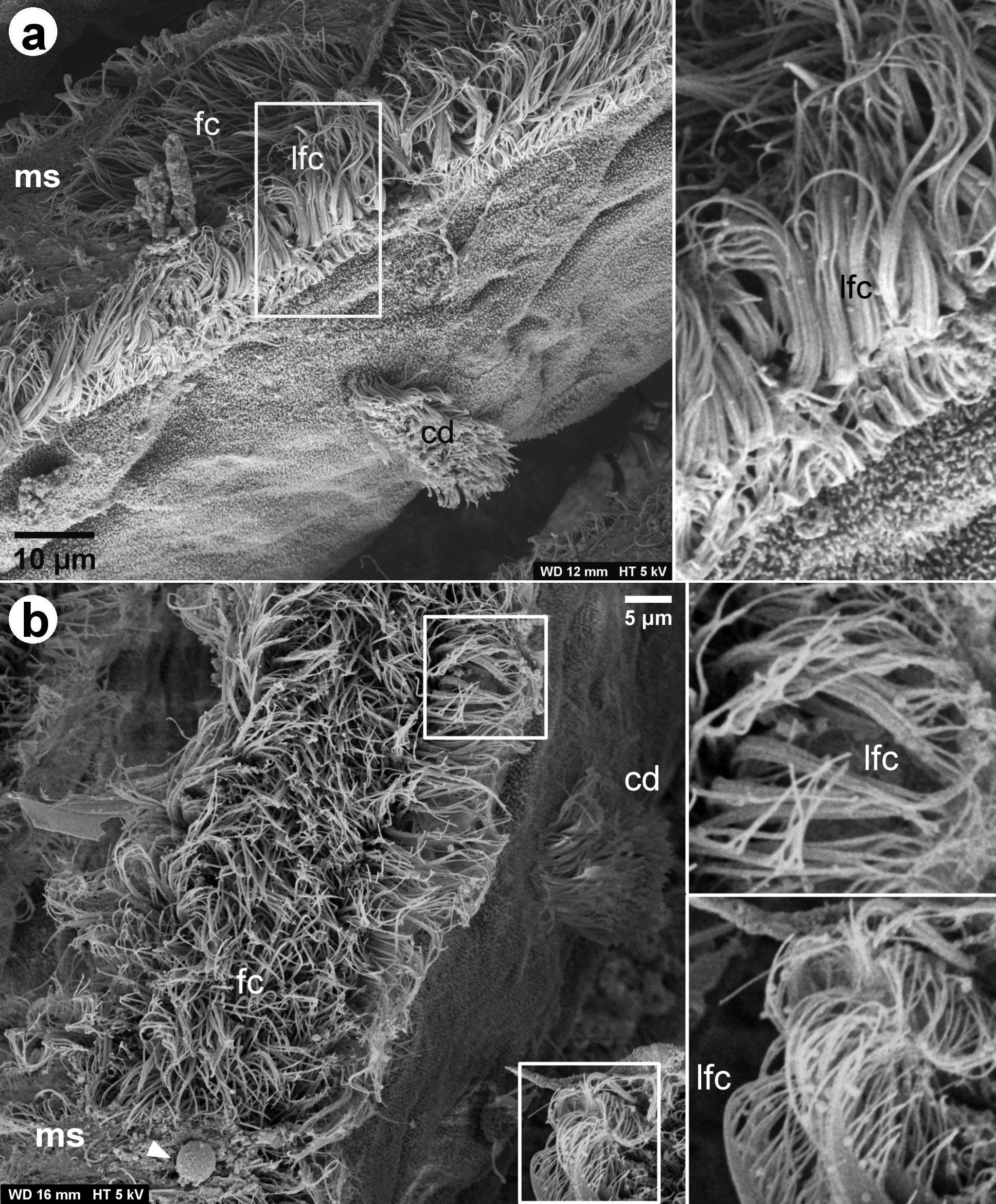

Supplement: Supplementary file 5 — Additional file 5. SEM images of the frontal and lateral tract of gillfilament. The white arrowhead points a 4-5 µm particle on the frontal tract.Legend: ms – mucus string, fc – frontal cilia, lfc – laterofrontal cirri, cd –ciliary dics. [file 40850_2022_107_MOESM5_ESM.tif]

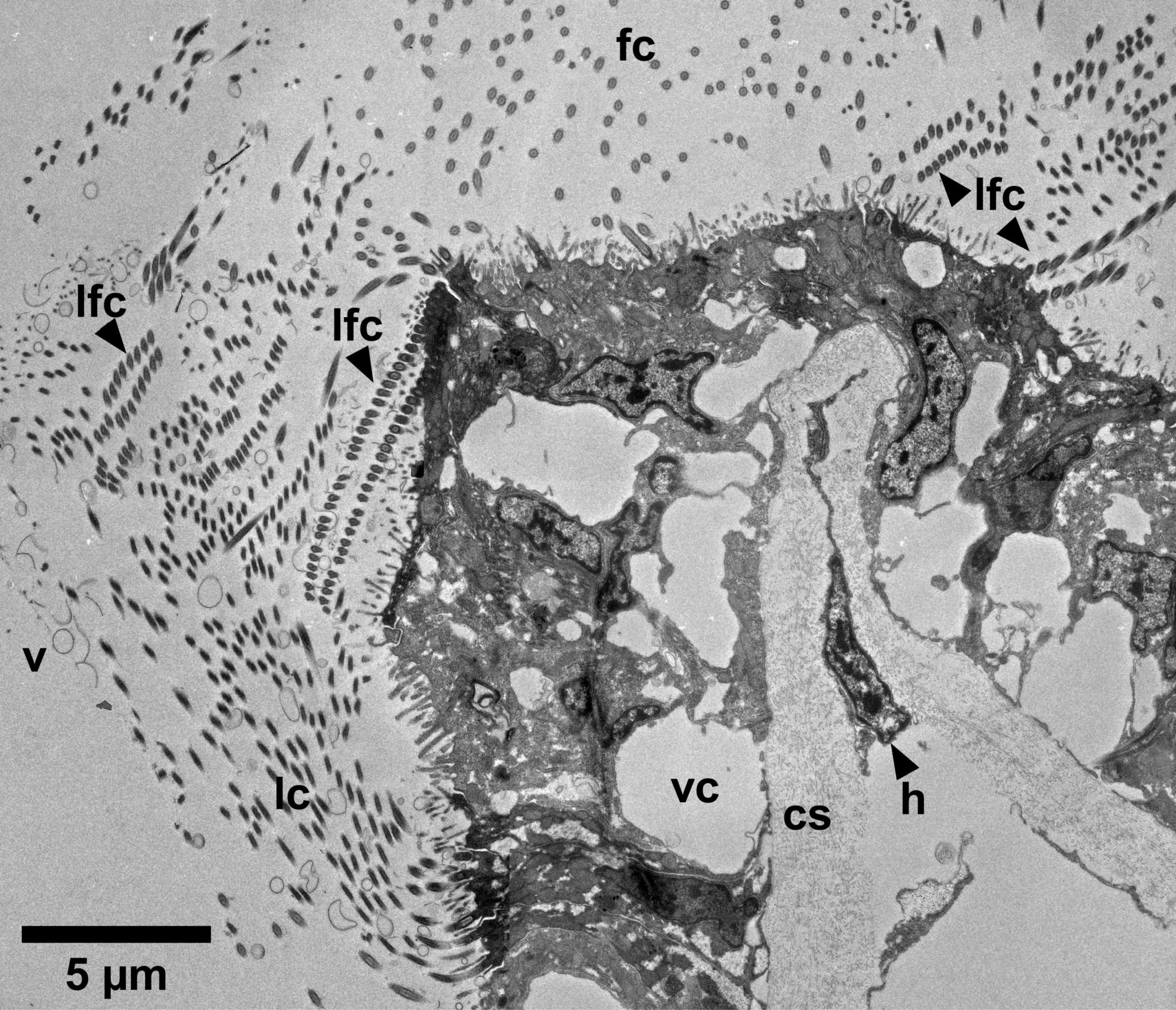

Supplement: Supplementary file 6 — Additional file 6. Montage of bright-field TEM images of a thin sectiontransversally to a gill filament of L.fortunei, showing its frontal portion. Lateral cilia [lc], laterofrontalcirri [lfc], and frontal cilia [fc] are observed in cross-section view.Spherical vesicles [v], vacuoles [vc], the collagenous structure [cs] of the hemolymph,and a hemocytes [h] are indicated. [file 40850_2022_107_MOESM6_ESM.tif]

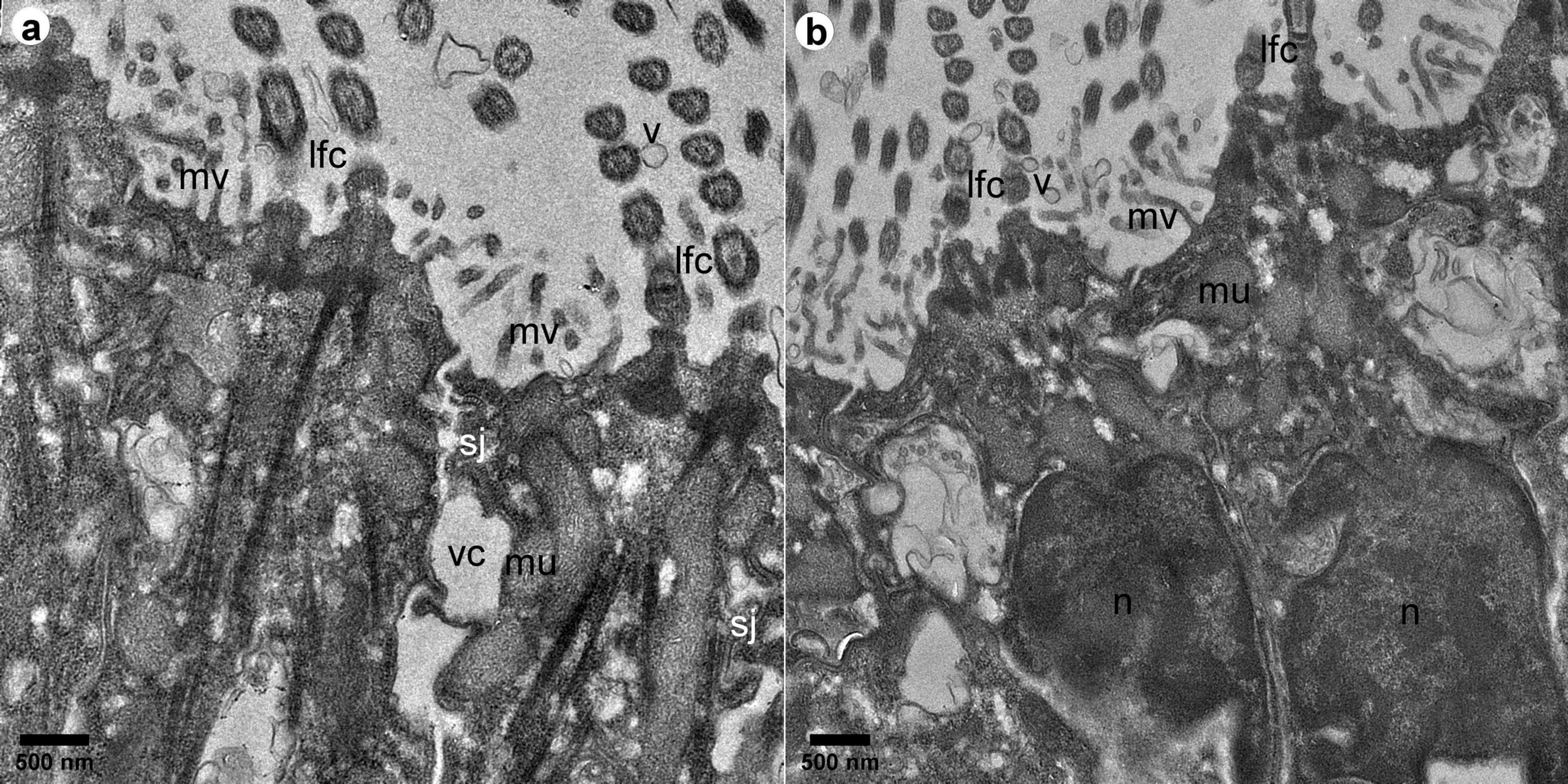

Supplement: Supplementary file 8 — Additional file 8. Bright-field TEM images showing coronal thin sectionsof the gill epithelium of L. fortunei.The laterofrontal cirri [lfc] are shown in cross-section view. Mucins [mu] andvacuoles [vc] are observed in the cells that possess microvilli [mv]. TSphericalvesicles [v] are present close to the lfc and septate junctions [sj] and nuclei[n] are indicated. [file 40850_2022_107_MOESM8_ESM.tif]

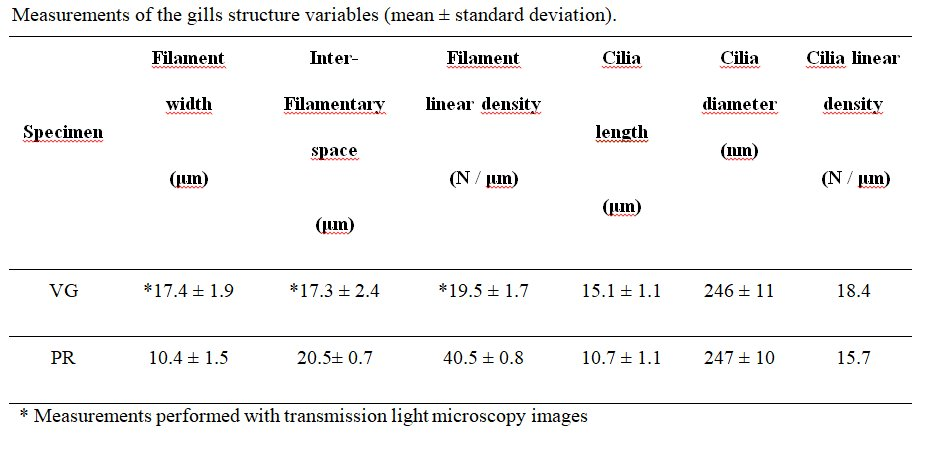

Supplement: Supplementary file 9 — Additional file 9. Measurements of the gills structure variables (mean ±standard deviation) of the Volta Grade (VG) and Paranaíba River (PR) specimens.Errors were estimated from measurements performed in different parts of theimage. [file 40850_2022_107_MOESM9_ESM.tif]
